# Disease Modification of Pustular Psoriasis by Secukinumab: A 20‐Year Follow‐Up of Von Zumbusch‐Type Generalized Pustular Psoriasis With Evolution Into Annular Pustular Psoriasis—A Case Report

**DOI:** 10.1002/kjm2.70083

**Published:** 2025-08-12

**Authors:** Yu‐Han Alice Hsu, Cheng‐Che E. Lan, Sheng‐Yiao Lin

**Affiliations:** ^1^ Department of Dermatology Kaohsiung Medical University Hospital Kaohsiung Taiwan; ^2^ Department of Dermatology, College of Medicine Kaohsiung Medical University Kaohsiung Taiwan

A 45‐year‐old male with von Zumbusch‐type generalized pustular psoriasis (GPP), diagnosed at age 20, was admitted to our ward with presentation of generalized tiny pustules and erythema with fever and illness during a flare‐up in 2005 (Figure 1A). Despite treatment with cyclosporine, methotrexate, and acitretin, the patient continued to experience recurrent exacerbations. In June 2017, he started treatment with Secukinumab (300 mg at baseline, weeks 1–4, then monthly) and daily acitretin 25 mg, and his skin lesions transitioned from erythema to annular hyperkeratotic plaques with pustules along the edges (Figure 1B). His condition remained stable; however, after discontinuing Secukinumab in July 2023, he experienced a flare‐up in November 2023. Compared to 2017, the pustular plaques were more well‐demarcated with a poly‐annular pattern, characteristic of typical annular pustular psoriasis lesions (Figure 1C,D). There were no symptoms of fever or fatigue. A skin biopsy confirmed pustular psoriasis with psoriasiform acanthosis, subcorneal microabscesses, and proliferated vessels in dermal papillae (Figure 1E,F). Secukinumab was introduced on day 14 of his admission, resulting in significant improvement. The pustules subsided, and skin plaques began desquamating with resolution within a time span of 1 week (Figure 1G). The patient was subsequently discharged with follow‐up at the outpatient department. Afterwards, the patient continued Secukinumab 300 mg monthly, achieving complete clearance for 18 months.

**FIGURE 1:**
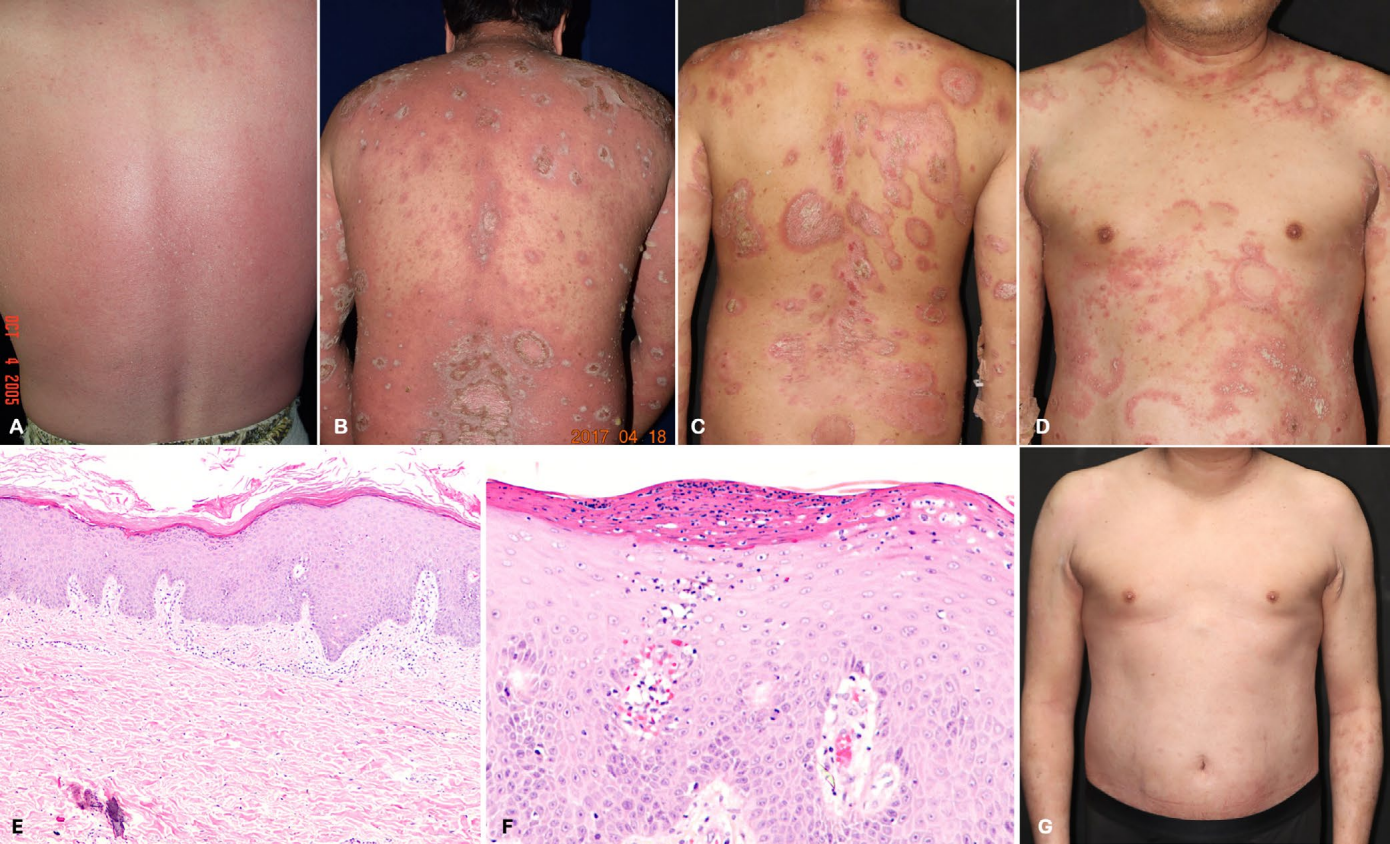
Generalized tiny pustules with erythema (A). Multiple erythematous, annular shape‐like hyperkeratotic plaques with pustules along the edges after the first dose of Secukinumab (B). Multiple poly‐annular and circular pattern pustular plaques, presenting typical annular pustular psoriasis lesions after cessation of Secukinumab (C) and (D). Biopsy from the trunk showed psoriasiform acanthosis, parakeratosis, and proliferated vessels in dermal papillae with inflammatory infiltration (hematoxylin–eosin stain, original magnification ×40) (E). Subcorneal neutrophilic microabscesses (hematoxylin–eosin stain, original magnification ×40) (F). Annular pustular plaque subsided with post‐inflammatory pigmentation after Secukinumab prescription (G).

Pustular psoriasis is classified into four types: von Zumbusch‐type GPP, annular pustular psoriasis (APP), exanthematic pustular psoriasis, and localized pustular psoriasis. GPP is characterized by erythematous plaques and pustules, with systemic symptoms like fever, malaise, neutrophilia, elevated C‐reactive protein, hypocalcemia, and abnormal liver function. Life‐threatening complications like sepsis and organ failure can occur [1]. On the other hand, APP is less acute and a more benign variant, characterized by annular or serpiginous lesions with pustules on the edges without systemic involvement [2], with treatment depending on severity. Localized cases use topical therapies like corticosteroids and retinoids, while moderate‐to‐severe cases require systemic treatments such as the application of methotrexate, cyclosporine, acitretin, and biologics. Secukinumab, an IL‐17A monoclonal antibody, effectively reduces inflammation, keratinocyte proliferation, and neutrophil recruitment in GPP.

In this patient with long‐standing Von Zumbusch‐type GPP, Secukinumab modified the disease, transforming it into a less severe form resembling APP, without systemic symptoms. This suggests Secukinumab's potential to reduce severity. Although no reports link Von Zumbusch GPP to APP, Liao et al. noted that severe juvenile‐onset APP unresponsive to treatment can progress to GPP [3]. Secukinumab has successfully treated APP in other cases. Khosravi‐Hafshejani et al. reported sustained clearance in a woman with chronic annular pustular psoriasis [4]. Herrero‐Moyano et al. also documented significant improvement in a woman with severe pustular eruptions after Secukinumab treatment [5].

In conclusion, after a 20‐year follow‐up of a patient with pustular psoriasis, Secukinumab modified the disease course of the severe form of Von Zumbusch‐type GPP to a more benign form of APP. Further studies are needed to investigate the pathogenesis behind the transformation of pustular psoriasis variants and to evaluate the effectiveness of Secukinumab in disease modification of GPP.

## Conflicts of Interest

The authors declare no conflicts of interest.

## Data Availability

The data that support the findings of this study are available on request from the corresponding author. The data are not publicly available due to privacy or ethical restrictions.
